# Spontaneous Expulsion of a Huge Cervical Fibroid After Uterine Artery Embolization Done as a Lifesaving Procedure for Acute Severe Abnormal Uterine Bleeding

**DOI:** 10.7759/cureus.30163

**Published:** 2022-10-11

**Authors:** Sharmeen I Memon, Neema S Acharya, Jyotsana Potdar

**Affiliations:** 1 Department of Obstetrics and Gynaecology, Jawaharlal Nehru Medical College, Datta Meghe Institute of Medical Sciences, Wardha, IND

**Keywords:** severe anemia, leiomyoma, abnormal uterine bleeding, uterine artery embolization, uterine fibroid

## Abstract

Uterine fibroids are a prevalent disease that most commonly affects women of reproductive age group and causes symptoms such as abnormal uterine bleeding that can have a detrimental impact on their quality of life. Being in the reproductive age group, fertility-conserving modalities in the form of hormonal therapy, myomectomy, hysterectomy, and uterine artery embolization (UAE) remain the main treatment options. Uterine artery embolization is one of the treatment options for select cases of fibroid uterus. Here, we present the case of a 36-year-old woman diagnosed with cervical leiomyoma who presented with uncontrollable uterine bleeding, severe anemia, and many failed medical therapies. Although the case did not fit within the usual UAE guidelines, the treatment was done to tide over the crisis as a temporary method to control severe hemorrhage. Bleeding was controlled immediately and the patient was getting prepared for major surgery with the correction of severe anemia. However, seven days after UAE, the fibroid spontaneously expelled itself, resulting in improved symptoms and the avoidance of surgery. The patient is currently symptomless and has improved sexual life, self-esteem, and quality of life.

## Introduction

Uterine leiomyomas (fibroids) are one of the most commonly occurring benign smooth muscle tumors in women, with more than 95% occurring in the uterine body. Cervical fibroids are extremely rare, accounting for fewer than 5% of uterine myomas, with around 50% being symptomatic [1,2]. They are usually asymptomatic but can cause abnormal uterine bleeding (AUB), chronic pelvic pain, dysmenorrhea, pressure-related symptoms, and infertility [3]. Conservative therapy options, such as myomectomy, can be considered in patients planning to conceive in the future. Hysterectomy and myomectomy, on the other hand, are linked to a longer hospital stay, a higher risk of blood loss, a longer operating time, and postoperative problems, all of which are concerning [4,5].

Surgical treatment of cervical fibroids necessitates a high level of surgical expertise. The risks are associated with the exact location of the cervical fibroid and poor cervical flexibility, which results in a limited approach to the operating field and difficulty in suturing, as well as an increased risk of intraoperative hemorrhage and potential injuries to the adjacent organs [6]. Because there is no uniform surgical treatment technique for cervical fibroids, management is based mostly on the patient’s presentation, the desire for future conception, and protocols of specific centers and surgeons [7].

Uterine artery embolization (UAE) has been the treatment option for symptomatic uterine fibroids in select women who want to have a future pregnancy [8].

## Case presentation

A 36-year-old, para two, live two, experiencing heavy menstrual bleeding associated with clot passage and dysmenorrhea for the last three years, with a considerable increase in the severity in the last 15 days, presented to the Gynaecology Outpatient Department. A pulse rate of 120 beats per minute (tachycardia), blood pressure of 100/60 mmHg, and temperature of 37.1°C were the vital signs on admission. Clinical examination was insignificant except for distinct pallor. The results of the cardiovascular and respiratory examination were within normal limits. There was no tenderness, guarding, rigidity, or discomfort in the abdomen.

On gynecological examination, the cervix was not visible per speculum; however, a cervical polyp/fibroid measuring 15 × 10 cm was found in the vagina which bled when touched. A similar finding of a 15 × 10 cm polyp/fibroid arising from the cervical canal was palpable on vaginal examination, firm in consistency, and the cervical rim could be palpated circumferentially around the polyp/fibroid. The uterus was palpated above the mass and was found to be normal in size, anteverted/anteflexed. A uterine sound test revealed that the uterocervical length was 3.5 inches.

**Figure 1:**
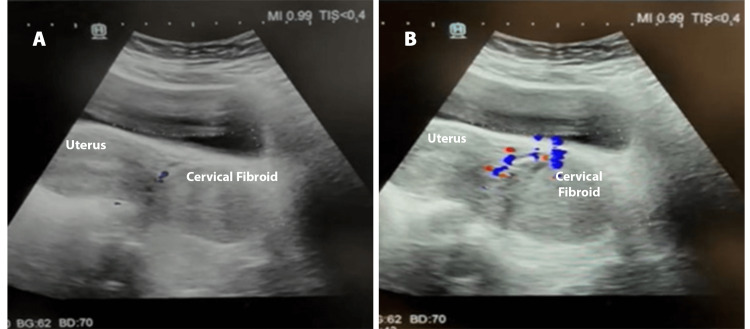
Ultrasound of the pelvis with Doppler. (A) A large cervical fibroid measuring 14.5 × 10.5 cm. (B) Cervical fibroid showing marked vascularity.

A well-defined, heterogeneously hypoechoic lesion originating from the cervix and measuring 14.5 × 10.5 cm was found on an ultrasound of the pelvis. Color Doppler revealed significant vascularity of the lesion. The uterus and bilateral adnexa were normal. The findings confirmed a cervical fibroid (Figures 1A, 1B).

She had severe anemia (hemoglobin = 4 g%). Three units of blood were transfused. The patient continued to bleed excessively despite ongoing medical care and anemia correction. After the initial resuscitation of the patient, the case was discussed with an intervention radiologist, and bilateral uterine artery embolization (UAE) was considered as an interim procedure until anemia correction and anesthetic fitness for major surgery was obtained.

**Figure 2:**
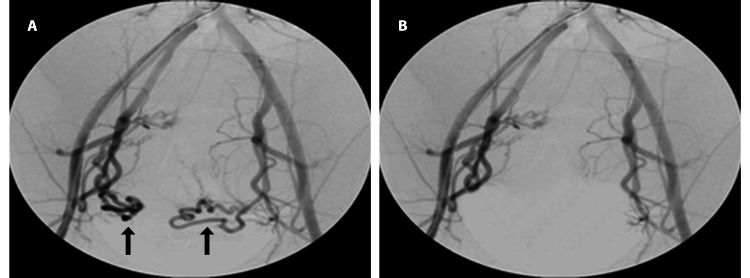
Digital subtraction angiography images during the uterine artery embolization procedure. (A) Pre-embolization images showing dilated and tortuous uterine arteries (black arrows). (B) Post-embolization images showing effective embolization of the uterine arteries.

The patient successfully underwent percutaneous bilateral UAE via the right common femoral artery route under all aseptic precautions. A Terumo Progreat microcatheter was used to selectively cannulate the uterine artery. The feeders supplying the fibroid were occluded with polyvinyl alcohol particles ranging in size from 300 to 500 nm. The treatment proceeded uneventfully when the angiogram revealed no flow within the fibroid (Figures 2A, 2B).

**Figure 3:**
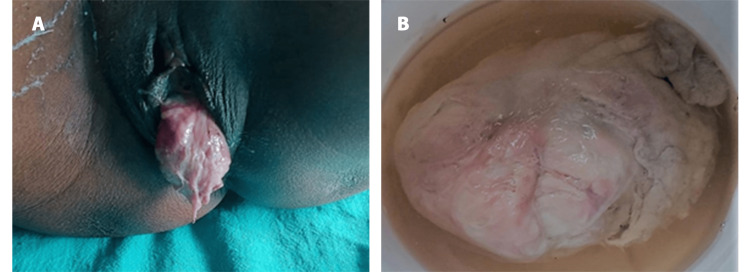
Huge cervical fibroid expulsion. (A) Spontaneous expulsion through the vagina (B). Gross specimen measuring 15 × 10cm in size.

After UAE, the patient’s symptoms significantly improved. The patient was scheduled for myomectomy; however, seven days after UAE, the fibroid expelled spontaneously through the vagina, as shown in Figures 3A, 3B. After the expulsion, there was no active bleeding. The patient’s hemodynamics were stable, which led to the avoidance of a major surgery like a myomectomy. Histopathological examination of the cervical fibroid revealed characteristics consistent with a leiomyoma.

The patient was discharged from the hospital, and after six weeks, her symptoms subsided, her hemoglobin level was 11.2 g%, and she was extremely pleased with her overall experience.

## Discussion

UAE as a treatment modality for uterine fibroids is primarily performed by an interventional radiologist. In this, the uterine arteries are embolized using polyvinyl alcohol particles of trisacryl gelatin microspheres via a transcutaneous femoral artery approach, which results in the devascularization and involution of the fibroid [9]. UAE is a relatively secure and efficient non-surgical fibroid treatment option, but rare consequences such as sepsis have been documented.

Post-embolization syndrome, which manifests as a low-grade temperature, pain, exhaustion, vomiting, and nausea, is a common minor consequence. Abnormal uterine and ovarian failure, as well as mass expulsion, and vaginal discharge are all modest problems [10]. Although fibroid ischemia after UAE can result in symptomatic improvement, it is linked to a higher chance of fibroid expulsion, as seen in this case. The treatment of fibroid expulsion and subsequent infection should be tailored to the patient’s clinical status and the avoidance of septic shock should be considered.

In this case, the patient had fibroid expulsion seven days after UAE, with symptom relief and no need for additional surgical procedures, demonstrating the efficacy of UAE. According to the available literature, fibroid expulsion after UAE was reported in 5-15% of cases, with time periods ranging from days to years [11].

Outcomes of UAE with myomectomy and hysterectomy have been compared in different randomized control clinical trials. UAE led to a shorter length of hospital stay, speedy healing, and quick resumption to daily normal activities, but it also resulted in a higher rate of mild complications. Moreover, research has been conducted revealing that patients who underwent UAE experienced considerably less blood loss than people who underwent a hysterectomy. Minor problems, such as fever, discomfort, and vaginal discharge, were observed to become increasingly prevalent in women undergoing UAE in comparison to others who underwent a hysterectomy [12]. The occurrence of so-called severe consequences such as pulmonary embolism and sepsis is quite low. Comparing hysterectomy and myomectomy groups, one case-control research found that the UAE group had a higher readmission and reoperation rate. Fertility after UAE demonstrated that pregnancy is possible with ovarian function and preserved uterine volume. Individualized UAE as an indication for females with symptomatic fibroids presenting with AUB who intend to conceive in the near future is recommended [13].

## Conclusions

UAE, used in the vast majority of symptomatic leiomyomas, is an effective, safe, and well-tolerated alternative to surgery. It is an excellent alternative for females who wish to keep their uterus. It is associated with a low complication rate, excellent clinical outcomes, and high patient satisfaction rates. Every effort should be made to make women who are experiencing fibroid-related symptoms aware of UAE as a treatment option.
